# DOMICILIARY SERVICES IMPROVE ACCESS TO DENTAL CARE FOR FUNCTIONALLY DEPENDENT OLDER ADULTS

**DOI:** 10.1093/geroni/igz038.1825

**Published:** 2019-11-08

**Authors:** Mathew A Lim, Gelsomina L Borromeo

**Affiliations:** Melbourne Dental School, University of Melbourne, Melbourne, Victoria, Australia

## Abstract

There is growing evidence demonstrating links between oral diseases and general health. The increased retention of teeth among functionally-dependent older adults presents a unique challenge in maintaining the oral health of these individuals from basic oral hygiene to accessing dental services. The results of our cross-sectional study demonstrate the important role domiciliary dental services play in reducing the barriers to accessing oral health care in this cohort. In our study, most individuals treated by domiciliary services lived in residential aged care facilities and were significantly older than those treated by hospital and community-based dental services dedicated to the specialized care of individuals with additional health care needs. A significantly higher number of those receiving domiciliary care were unable to self-consent for treatment compared to those managed in other settings. 27.4% of these patients had a diagnosis of dementia. More than half (56.9%) of patients treated by domiciliary services received some form of treatment with almost half (48.1%) of these requiring a dental extraction. Only two of these patients were not diagnosed with a chronic condition known to affect oral health (dementia, Parkinson’s disease, diabetes mellitus, arthritis, stroke, osteoporosis). 23.7% of domiciliary appointments were used for denture fabrication. The results depict the worrying level of unmet treatment need in residents of aged care facilities. However, they also demonstrate the potential for domiciliary dental services to play a role in developing partnerships between carers and oral health professionals to improve the oral health of functionally-dependent older adults.

